# Routes of Cancer Dissemination: Distinguishing Lymphatic and Hematogenous Spread from Venous Entry to Systemic Arterial Distribution

**DOI:** 10.3390/cancers18142256

**Published:** 2026-07-14

**Authors:** Stanley P. Leong

**Affiliations:** 1 California Pacific Medical Center, San Francisco, CA 94107, USA; stanley.leong@sutterhealth.org; 2 University of California, San Francisco School of Medicine, San Francisco, CA 94143, USA

**Keywords:** cancer metastasis, cancer dissemination, sentinel lymph node (SLN), lymphatic dissemination, hematogenous dissemination, arterial circulation, post-capillary venules, lymphangiogenesis, pre-metastatic niche, organ tropism

## Abstract

**Background/Objectives:** Cancer metastasis is responsible for most cancer-related deaths, yet the precise anatomical and physiological routes by which cancer cells disseminate remain incompletely defined. This review aims to present an integrated model of lymphatic and hematogenous dissemination that provides a unified framework for understanding metastatic progression. **Methods:** The published literature on lymphatic biology, microvascular physiology, tumor immunology, and cancer metastasis was critically reviewed and integrated to develop a comprehensive anatomical and physiological model of cancer dissemination. **Results:** The proposed model identifies lymphatic dissemination as the predominant metastatic route in many solid tumors. Cancer cells enter structurally permissive initial lymphatic capillaries and are transported to the sentinel lymph node (SLN), where interactions with the tumor microenvironment may eliminate disseminated cells, maintain dormancy, or facilitate immune escape and further dissemination. Cancer cells that survive within or escape beyond the SLN subsequently travel through collecting lymphatics and the thoracic or right lymphatic duct to enter the systemic venous circulation. Following cardiopulmonary transit, surviving cells may be redistributed through the systemic arterial circulation to distant organs. A secondary pathway involves direct hematogenous intravasation through post-capillary venules, where reduced shear stress, increased endothelial permeability, and permissive endothelial biology facilitate entry into the venous circulation. Thus, lymphatic and direct venular pathways ultimately converge in the venous circulation before systemic arterial dissemination. **Conclusions:** This unified model integrates lymphatic and hematogenous dissemination into a coherent anatomical and physiological framework. By emphasizing the SLN as an early immunologic checkpoint and the arterial circulation as the final distribution network for disseminated cancer cells, this review provides a conceptual basis for understanding metastatic patterns and identifying biomarkers and therapeutic vulnerabilities.

## Simple Summary

Cancer metastasis is the principal cause of cancer-related mortality, yet the precise anatomical and physiological routes of cancer dissemination remain incompletely defined. This review proposes a unified model in which lymphatic dissemination represents the predominant metastatic pathway for many solid tumors. Cancer cells enter structurally permissive initial lymphatic capillaries and are transported to the sentinel lymph node (SLN), where immune and stromal interactions may eliminate disseminated cells, maintain dormancy, or facilitate immune escape and further progression. Surviving cells may subsequently travel through efferent lymphatics, higher echelon lymph nodes, and the thoracic or right lymphatic duct into the systemic venous circulation, pass through the cardiopulmonary circulation, and are distributed by the systemic arterial circulation to distant organs. A secondary pathway involves direct hematogenous intravasation through post-capillary venules before converging with the same systemic circulation. This integrated framework provides new insight into metastatic progression, identifies the SLN as an early immunologic checkpoint, and highlights potential biomarkers and therapeutic vulnerabilities.

## 1. Introduction

Cancer metastasis is a complex, multistep biological process that accounts for the majority of cancer-related deaths. Since the Seed and Soil Hypothesis was first proposed by Stephen Paget in 1889, emphasizing the compatibility between disseminated cancer cells and the host organ microenvironment [1,2], substantial effort has been devoted to elucidating the molecular and cellular mechanisms underlying metastatic progression [3,4,5,6]. In contrast, the precise anatomical and physiological routes by which cancer cells disseminate through the lymphatic and vascular systems have received comparatively less attention.

**Figure 1:**
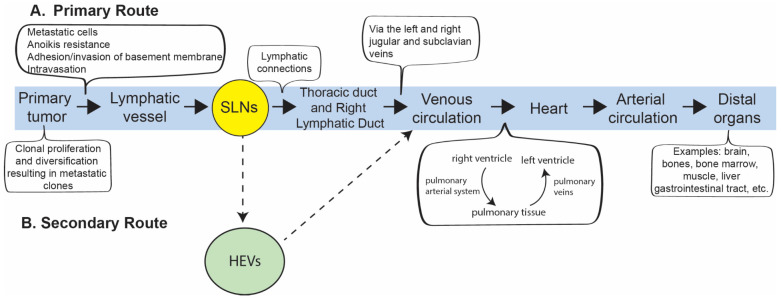
Routes of Cancer Dissemination: Distinguishing Lymphatic and Hematogenous Spread from Venous Entry to Systemic Arterial Distribution. Cancer cells may disseminate from the primary tumor through afferent lymphatics to the sentinel lymph node(s) (SLN[s]) and then through efferent lymphatics, higher-echelon lymph nodes, and the thoracic or right lymphatic duct into the systemic venous circulation. After cardiopulmonary transit, surviving cells may enter the systemic arterial circulation and be distributed to distant organs. A secondary hematogenous route involves direct cancer-cell entry through post-capillary venules at the primary tumor site or metastatic deposits. In tumor-draining lymph nodes, high endothelial venules (HEVs) represent specialized post-capillary venules that may serve as a nodal vascular interface. Thus, HEVs are shown as a specialized nodal venular compartment, whereas post-capillary venules represent the broader route for hematogenous intravasation. The molecular mechanisms of these routes of cancer dissemination are discussed in detail in the text.

In this review, we present an integrated model that distinguishes lymphatic dissemination through the SLN(s), efferent lymphatics, and the thoracic or right lymphatic duct from direct hematogenous intravasation through post-capillary venules, while emphasizing that both pathways ultimately converge in the venous circulation and subsequently distribute cancer cells to distant organs through the systemic arterial circulation (Figure 1). Current evidence indicates that hematogenous dissemination is initiated predominantly through post-capillary venules rather than arterial vessels, reflecting major differences in vascular structure, hemodynamics, and endothelial permeability, and leukocyte-like trafficking mechanisms between the venular and arterial segments of the circulation [4,7].

## 2. Lymphatic Dissemination Through the SLN and Thoracic Duct/Right Lymphatic Duct into the Venous and Arterial Circulation

Initial lymphatic capillaries, situated within the interstitial compartment adjacent to the blood microcirculation at the primary tumor site, provide a highly permissive pathway for cancer cell entry because of their specialized structural and functional characteristics [8,9,10,11,12]. These blind-ended vessels are composed of overlapping lymphatic endothelial cells connected by discontinuous “button-like” junctions and are supported by a minimal or discontinuous basement membrane with little to no pericyte coverage. Together with their extremely low intraluminal hydrostatic pressure (approximately 0–4 mmHg), these features facilitate the relatively passive uptake of interstitial fluid, macromolecules, leukocytes, and cancer cells into the lymphatic circulation [8,10,13].

Following entry into the initial lymphatic capillaries, lymph and any entrained cancer cells are transported sequentially through precollecting and collecting lymphatic vessels, afferent lymphatics to the SLN, efferent lymphatics, higher-echelon lymph nodes, and larger lymphatic trunks. It should be noted that cancer-cell entry into the lymphatic system occurs primarily at the level of initial lymphatic capillaries, rather than through direct entry into downstream collecting lymphatics, lymphatic trunks, or central lymphatic ducts. Lymph from the right side of the head and neck, right upper extremity, and right hemithorax drains into the right lymphatic duct, whereas lymph from the remainder of the body drains into the thoracic duct. These central lymphatic ducts empty into the right and left venous angles, respectively, at the junctions of the internal jugular and subclavian veins. From these sites, cancer cells enter the right and left brachiocephalic veins, which unite to form the superior vena cava and return blood to the right atrium. Through cardiopulmonary transit, cancer cells pass through the right ventricle and pulmonary circulation, return via the pulmonary veins to the left atrium and left ventricle, and are subsequently ejected into the systemic arterial circulation, which distributes them to distant organs [3,13,14,15,16] (Figure 1).

## 3. Lymphangiogenesis and Formation of a Pre-Metastatic Niche

Cancer-associated lymphangiogenesis is a critical early event in metastatic progression that expands both peritumoral and nodal lymphatic networks and facilitates the transport of cancer cells and tumor-derived mediators to the SLN. This process is driven predominantly by vascular endothelial growth factor-C (VEGF-C) and VEGF-D, which bind to vascular endothelial growth factor receptor-3 (VEGFR-3; FLT4) expressed on lymphatic endothelial cells. Activation of this signaling pathway stimulates proliferation, migration, and remodeling of lymphatic vessels, resulting in increased lymphatic vessel density, enlargement of lymphatic channels, and enhanced lymph flow from the primary tumor to regional lymph nodes [9,11,17,18]. In many solid tumors, including melanoma and breast cancer, increased expression of lymphangiogenic growth factors and lymphatic endothelial markers—including VEGF-C, VEGF-D, LYVE-1, Podoplanin, and PROX1—has been associated with enhanced lymphangiogenesis, sentinel lymph node metastasis, increased recurrence, and poorer clinical outcomes [9,18,19,20]. The biological significance of lymphangiogenesis extends beyond the formation of additional conduits for tumor cell transport. Newly formed lymphatic vessels increase the surface area available for tumor cell entry and facilitate the delivery of soluble factors released by the primary tumor, including cytokines, chemokines, growth factors, extracellular vesicles, and exosomes. These mediators can reach the SLN before the arrival of viable cancer cells and initiate a cascade of molecular and cellular changes that condition the nodal microenvironment for future metastatic colonization [21,22,23]. Thus, the SLN is not merely a passive recipient of metastatic cells but an actively remodeled organ whose structure and immune function are altered in anticipation of the arrival of tumor cells.

Within the SLN, tumor-derived signals induce expansion of subcapsular and medullary lymphatic sinuses, remodeling of the extracellular matrix, and alterations in the architecture of high endothelial venules and fibroblastic reticular cell networks. These structural changes are accompanied by profound immunologic reprogramming involving dendritic cells, T lymphocytes, B-cell follicles, macrophages, and stromal cells. The resulting microenvironment often exhibits increased numbers of regulatory T cells, myeloid-derived suppressor cells, and alternatively activated macrophages, together with reduced antigen presentation and impaired cytotoxic T-cell activity [23,24,25]. These observations underscore the dual role of the SLN as both an immunologic barrier and a biologically permissive niche. Beyond serving as conduits for cancer-cell transport [26], lymphatic vessels actively participate in the regulation of anti-tumor immunity. Tumor-associated lymphatics facilitate the transport of tumor antigens from peripheral tissues to regional lymph nodes, promote dendritic-cell migration, and influence T-cell priming and activation within the SLN. Consequently, lymphatic vessels contribute not only to metastatic dissemination but also to immune surveillance and the generation of adaptive immune responses [27,28]. Recent studies in melanoma have further demonstrated that lymphatic remodeling alters local and regional immune parameters and may influence responsiveness to immunotherapy. Increased lymphangiogenesis can enhance antigen delivery and T-cell infiltration under certain conditions, indicating that lymphatic vessels may exert both pro-metastatic and anti-tumor functions depending on the biological context [27,28]. In some patients, effective anti-tumor immune surveillance within the SLN may eliminate disseminated cancer cells and prevent further metastatic spread. In others, tumor-induced lymphangiogenesis, stromal remodeling, and immune checkpoint-mediated suppression create a conditioned nodal microenvironment that supports cancer cell survival, immune escape, dormancy, and subsequent metastatic expansion [22,23,24]. This functional dichotomy forms the basis of the concept that the SLN acts simultaneously as a sentinel guard against cancer invasion or as an incubator [29] and gateway [26] to achieve subsequent dissemination.

Cancer cells that survive within the SLN may subsequently exit through efferent lymphatic vessels, traverse higher-echelon lymph nodes and collecting lymphatic trunks, then drain into the right lymphatic duct or the thoracic duct. These central lymphatic ducts empty into the jugular–subclavian venous junctions bilaterally, providing direct access to the systemic venous circulation. After passage through the right heart and pulmonary circulation, viable cancer cells enter the left heart and are redistributed through the systemic arterial circulation to distant organs, where they may arrest, extravasate, and establish metastatic colonies [3,14,16].

Taken together, lymphangiogenesis and pre-metastatic niche formation establish a direct mechanistic link between the primary tumor, the SLN, and distant metastasis. By expanding lymphatic access, transporting tumor-derived conditioning signals, and reprogramming the nodal microenvironment, this process may influence whether disseminated cancer cells are eliminated, maintained in dormancy, or enabled to progress toward systemic disease. Importantly, accumulating evidence indicates that lymphatic vessels function not merely as passive conduits for fluid and tumor-cell transport but also as active regulators of inflammatory and immune-cell niches. In melanoma, lymphatic vessels have been shown to regulate immune microenvironments, promote T-cell infiltration, and influence responsiveness to immunotherapy [27,28]. Experimental modulation of lymphangiogenesis can also alter local immune-cell composition, inflammatory signaling pathways, and tissue remodeling [30], further emphasizing the reciprocal relationship between lymphatic biology, immune regulation, and metastatic progression. Thus, the SLN serves not only as a critical staging landmark but also as a uniquely accessible model for investigating the earliest interactions between cancer cells and the host microenvironment and for identifying biomarkers and therapeutic vulnerabilities that govern metastatic progression.

## 4. Venular Intravasation and Hematogenous Spread

The predominant route by which cancer cells gain access to the systemic arterial circulation is through lymphatic dissemination to the SLN, followed by transport through the thoracic duct or right lymphatic duct into the venous circulation and subsequent cardiopulmonary transit as described above. A secondary route involves direct hematogenous intravasation through post-capillary venules, which provide the principal sites of cancer-cell entry into the bloodstream [3,7].

Post-capillary venules are uniquely suited for hematogenous dissemination because they combine relatively low hydrostatic pressure (approximately 10–15 mmHg), reduced shear stress, increased endothelial permeability, and abundant expression of adhesion molecules. In contrast, arteries and arterioles are resistant to tumor cell entry because of higher intraluminal pressure, greater shear forces, tightly organized endothelial junctions, and prominent smooth muscle layers [31,32]. To penetrate the venular wall, cancer cells exploit many of the same adhesion and signaling pathways that regulate leukocyte trafficking, including selectins, integrins, and members of the immunoglobulin superfamily [3,7,33]. Cancer cells and associated stromal and inflammatory cells also secrete vascular endothelial growth factor-A (VEGF-A), tumor necrosis factor-α (TNF-α), and angiopoietin-2, which increase endothelial permeability and disrupt intercellular junctions [7,34]. These changes facilitate venular intravasation at both the primary tumor site and established metastatic lesions, allowing cancer cells to re-enter the circulation and seed additional distant sites [7,33]. The process of entry through the post-capillary venular wall may be more efficient in the metastatic sites because the disseminated cells represent biologically selected subclones that have already survived circulation, immune surveillance, and adaptation to a permissive organ microenvironment [5]. Consequently, metastatic deposits can serve not only as end points of dissemination but also as secondary sources of cancer cells capable of re-entering the circulation and seeding additional metastases, a process termed metastasis-to-metastasis spread [35,36].

Lymphatic and venular intravasation differ fundamentally in both anatomy and mechanism. Initial lymphatic capillaries permit relatively passive uptake because of their discontinuous endothelial junctions, minimal basement membrane, sparse pericyte coverage, and extremely low intraluminal hydrostatic pressure as described above. By contrast, entry into post-capillary venules requires an active, multistep process involving endothelial adhesion, cytoskeletal remodeling, proteolytic degradation of the basement membrane, and regulated transendothelial migration across a more restrictive vascular barrier as described above [7,8,9,11].

Thus, lymphatic dissemination is favored by the inherently permissive architecture of initial lymphatic capillaries, whereas hematogenous dissemination is a highly regulated biological process that occurs predominantly at post-capillary venules. In both cases, access to arterial circulation ultimately depends on entry into the venous system, either indirectly through the lymphatic network or directly through the post-capillary venule (Figure 1).

## 5. Microcirculatory System and Architecture: Integrating Blood Exchange Capillaries, Post-Capillary Venules, and Lymphatic Capillary Drainage

**Figure 2:**
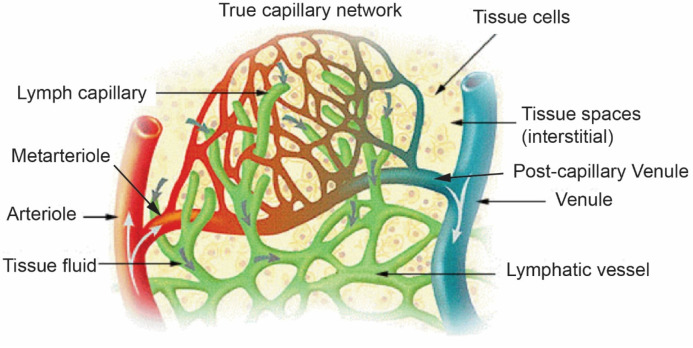
Microcirculatory Architecture: As an Integrated Continuum of Metarteriole, True Capillary Network, and Post-Capillary Venule. Schematic representation of the true capillary network (exchange capillary bed) that consists of a three-dimensional meshwork of interconnected endothelial tubes forming the anatomical bridge between the metarteriole and the post-capillary venule. From the heart, blood flows to the arteriole, then into the metarteriole, where the blood enters the capillary network, and then into the venous system via the post-capillary venule, and back to the heart. This image was modified from an image produced by SEER, Public domain, via Wikimedia Commons [40].

The microcirculatory system represents the intricate network of small blood vessels and lymphatic channels that support tissue viability throughout most of the body. This architecture includes terminal arterioles, metarterioles, true capillary networks, post-capillary venules, venules, arteriovenous shunts, and blind-ended lymphatic capillaries (Figure 2). Although microcirculation is fundamental to nearly all vascularized tissues, it is not literally present in every tissue microenvironment, because certain specialized structures are physiologically avascular, including the cornea, cartilage, and portions of the epidermis. In vascularized organs, however, the microcirculatory architecture is widely distributed and tissue-specialized, with organ-specific variation in vascular density, permeability, endothelial phenotype, perfusion pressure, immune-cell trafficking, and lymphatic drainage. Thus, the microcirculatory system should be understood as a near-universal but locally specialized anatomical and physiological interface between circulating blood, interstitial fluid, lymphatic drainage, and the surrounding tissue microenvironment [31,37,38,39].

An important implication of this widespread distribution is that each vascularized tissue microenvironment contains many terminal microvascular units, not a single unit. These repeated arteriolar–capillary–venular networks are distributed throughout tissues and organs, allowing circulating blood to continuously deliver oxygen and nutrients, exchange soluble factors, collect metabolic products, and return through the venous circulation. At the primary tumor site, blood flowing through tumor-associated microcirculatory networks may also collect cancer-derived materials such as cytokines, extracellular vesicles, cell-free nucleic acids, and tumor cells, before returning through the venous system to the cardiopulmonary circulation. This circulating blood may then pass through multiple downstream microvascular beds, where tumor-derived materials or circulating cancer cells encounter additional vascular interfaces. In this sense, the hematogenous route represents a continuously recirculating vascular pathway that can repeatedly connect the primary tumor with multiple organ microcirculatory beds through venous return, cardiopulmonary transit, and arterial redistribution. This differs anatomically and physiologically from the lymphatic route, in which tumor cells or tumor-derived material enter low-pressure lymphatic capillaries and pass through regional lymphatic channels, sentinel lymph nodes, and other regional lymph nodes before returning to the central venous system through the thoracic duct or right lymphatic duct and entering the cardiopulmonary circuit and systemic arterial distribution (Figure 1) [8,13,15,17,18,31,38,39,41].

The terminal microcirculation is organized as a continuous, highly specialized vascular unit comprising the metarteriole, the true capillary network, and the post-capillary venule (Figure 2). This microvascular continuum sustains normal tissue physiology while also providing the immediate microanatomical environment in which circulating cancer cells may undergo mechanical arrest, endothelial adhesion, extravasation, dormancy, or early metastatic colonization. Although different organs contain specialized vascular and lymphatic architectures, the detailed biological consequences of organ-specific tumor microenvironments are beyond the scope of this manuscript. In the present framework, the emphasis is on the anatomical and physiological routes through which tumor cells and tumor-derived materials move between lymphatic, venous, cardiopulmonary, arterial, and tissue microcirculatory compartments [3,7,16,31,42].

The metarteriole is a transitional vessel arising from the terminal arteriole and typically measures approximately 15–30 µm in diameter. Unlike larger arterioles, it contains discontinuous smooth muscle cells that form precapillary sphincters at the origins of selected capillary branches, thereby regulating blood flow into the downstream exchange network according to local metabolic demands. The metarteriole therefore serves as the functional gateway between the arterial circulation and the true capillary bed, modulating perfusion pressure, erythrocyte transit, and the distribution of blood flow through the microvascular network [39,43].

The true blood capillary network, also referred to as the blood exchange capillary bed, consists of a three-dimensional meshwork of interconnected endothelial tubes that forms the anatomical bridge between the metarteriole and the post-capillary venule (Figure 2). These vessels are blood capillaries and should be distinguished from adjacent blind-ended lymphatic capillaries in the interstitial space. Within the true blood capillary network, the arteriolar end receives blood from the metarteriole and is characterized by relatively higher hydrostatic pressure and greater filtration, whereas the venular end drains toward the post-capillary venule and is characterized by lower hydrostatic pressure and greater reabsorption. Individual true blood capillaries typically measure approximately 4–8 µm in diameter, closely approximating the diameter of erythrocytes, which often deform and traverse these vessels in single file. The capillary wall is composed of a single layer of endothelial cells supported by a basement membrane and scattered pericytes, without a smooth muscle layer. This minimal structure optimizes diffusion while maintaining the integrity of the blood–tissue barrier [3,7,39].

Functionally, the true capillary network is the principal site of oxygen delivery, carbon dioxide removal, nutrient exchange, hormone distribution, and metabolic waste clearance. Oxygen tension progressively declines along the capillary bed as oxygen diffuses from erythrocytes into surrounding tissues, whereas carbon dioxide tension increases as metabolic by-products enter the circulation. Hydrostatic pressure also declines from the arterial to the venous side of the exchange network, promoting filtration of plasma-derived fluid into the interstitial space and subsequent reabsorption into the venular side of the microcirculation or uptake by adjacent lymphatic capillaries [37,38,39,41].

This blood–interstitial–lymphatic interface is central to both normal tissue homeostasis and cancer dissemination. Approximately 20 L of plasma-derived fluid is filtered daily from systemic capillaries into the interstitial space, predominantly across the arteriolar and metarteriolar side of the exchange capillary network. Of this filtered volume, an estimated 15–18 L are subsequently reabsorbed into venular capillaries and post-capillary venules, whereas the remaining 2–4 L are taken up by lymphatic capillaries and returned to the venous circulation through collecting lymphatic vessels and central lymphatic ducts. Thus, the interstitial space functions as a central extracellular reservoir linking the blood and lymphatic compartments [13,15,37,38,41].

Hydrostatic pressure declines progressively across the systemic microcirculation, from approximately 35–50 mmHg in terminal arterioles to approximately 25–35 mmHg in metarterioles, approximately 30–35 mmHg at the arteriolar end of the true capillary network, approximately 10–15 mmHg at its venular end, approximately 10–15 mmHg in post-capillary venules, and approximately 5–10 mmHg in larger collecting venules. These values should be regarded as approximate physiological ranges rather than fixed constants, as they vary among tissues and according to local hemodynamic conditions. The key principle is the marked decline in hydrostatic pressure from the arterial to the venous side of the microcirculation, culminating in the relatively low-pressure environment of post-capillary venules. Lymphatic capillaries operate at even lower pressure, approximately 0–4 mmHg, creating a highly permissive environment for interstitial-fluid uptake, macromolecular clearance, immune-cell entry, and, in cancer, potential tumor-cell access to lymphatic drainage pathways [8,13,15,38,39,43,44].

The dimensions and flow properties of the true capillary network have important implications for metastatic dissemination. Most circulating cancer cells measure approximately 15–25 µm in diameter, substantially larger than the luminal diameter of exchange capillaries. As a result, cancer cells may undergo transient mechanical arrest, deformation, or fragmentation within the capillary meshwork, particularly in organs with dense microvascular beds such as the lung, liver, bone marrow, and brain. However, physical trapping alone does not necessarily constitute productive extravasation or metastatic colonization. Many arrested tumor cells undergo apoptosis, remain transiently lodged, or fail to adapt to the foreign tissue microenvironment [3,7,16,45].

The post-capillary venule, typically measuring approximately 10–30 µm in diameter, represents the first venous segment of the microcirculation and serves as the predominant physiological site of leukocyte trafficking. Compared with exchange capillaries, post-capillary venules possess a larger lumen, lower shear stress, increased endothelial permeability, and higher expression of selectins, integrins, chemokines, and immunoglobulin-superfamily adhesion molecules. These features create a uniquely permissive environment for regulated cell adhesion, endothelial interaction, and transendothelial migration. In cancer, these venular characteristics may facilitate tumor-cell adhesion, endothelial remodeling, and productive extravasation into the surrounding interstitial space [7,34,46].

Accordingly, a biologically plausible model is that circulating cancer cells may first undergo transient mechanical arrest within the true capillary network and then either deform through the capillary bed or become carried into downstream post-capillary venules, where the structural and molecular properties of the venular endothelium favor regulated extravasation. Once within the interstitial space, disseminated cancer cells may undergo apoptosis, enter dormancy, interact with immune and stromal cells, or establish proliferative colonies that eventually evolve into clinically detectable metastases. This process is shaped not only by vascular anatomy and hemodynamics, but also by endothelial activation, immune-cell trafficking, local inflammation, extracellular-matrix remodeling, and tissue-specific metastatic niches [7,16,23,42,45].

The lymphatic component of the microcirculatory architecture is equally important. Lymphatic capillaries are blind-ended, low-pressure channels with specialized endothelial junctions that allow entry of interstitial fluid, macromolecules, immune cells, and, under pathological conditions, tumor cells. In tissues where lymphatic drainage is prominent, malignant cells may enter lymphatic capillaries and migrate to regional lymph nodes, including the sentinel lymph node. This provides the anatomical basis for lymphatic dissemination in cancers such as melanoma [47] and breast cancer [48] and links local tumor biology to regional nodal metastasis, immune surveillance, and potential systemic progression [8,13,15,17,18].

Thus, the metarteriole, true capillary network, post-capillary venule, and lymphatic capillary system constitute an integrated microcirculatory continuum that maintains tissue perfusion, fluid homeostasis, immune surveillance, and tissue repair. In the context of cancer metastasis, this same architecture provides the anatomical and biological framework through which tumor cells are mechanically filtered, exposed to endothelial and immune selection, permitted to enter venular or lymphatic pathways, and ultimately enabled or prevented from colonizing distant or regional sites. This integrated view of the microcirculatory system is therefore essential for understanding the distinction between lymphatic dissemination, venular intravasation or extravasation, venous return, cardiopulmonary transit, arterial distribution, and organ-specific metastatic colonization (Figure 1).

## 6. Arterial Dissemination and Organ Tropism

After surviving transit through the right heart and pulmonary circulation, circulating cancer cells enter the left atrium and left ventricle and are ejected into the systemic arterial circulation, which distributes them throughout the body. At this stage, dissemination becomes a process of arterial redistribution in which cancer cells are delivered to the terminal microvascular beds of distant organs, including the brain, bone marrow, liver, lung, adrenal glands, and skeletal muscle. Although this distribution follows established patterns of blood flow, the eventual sites of metastasis are highly selective and reflect the interaction between hemodynamic delivery and the capacity of cancer cells to survive and adapt within specific tissue microenvironments [3,4,49].

The first step in organ colonization is governed in part by anatomical and vascular constraints. Cancer cells must traverse the arterial circulation and become arrested within the terminal microcirculation (Figure 2), where vessel diameter, endothelial phenotype, and local flow characteristics influence their retention. Different primary tumors at different anatomical sites encounter distinct first-pass vascular filters depending on their venous drainage. Cancers arising within the gastrointestinal tract commonly disseminate through the portal venous system and are delivered first to the liver, helping to explain the high frequency of hepatic metastases in colorectal cancer. In contrast, melanoma and breast cancer tend to drain into the lymphatic system and then the venous circulation, with an increased incidence of lymph node involvement. Many sarcomas commonly encounter the pulmonary capillary bed as the first major vascular filter, contributing to the high incidence of lung metastases [3,16].

Mechanical delivery alone does not fully explain patterns of metastasis. For example, skeletal muscle [50], the spleen [51], and the heart, particularly the myocardium [52], receive substantial blood flow yet are relatively uncommon sites of metastatic colonization. These observations indicate that successful metastasis depends not only on vascular delivery but also on the compatibility of disseminated cancer cells with the organ-specific endothelial, stromal, metabolic, and immune microenvironment, consistent with Stephen Paget’s Seed and Soil Hypothesis [1,3,49].

The concept of organ tropism is most clearly illustrated by recurring clinical patterns of metastasis. Melanoma commonly spreads to regional lymph nodes and distant sites, including the lung, liver, brain, and small bowel [53,54]. Regarding distant organs, breast cancer exhibits a strong predilection for bone, lung, liver, and brain [55,56]. Prostate cancer characteristically metastasizes to bone, particularly the axial skeleton [35,57]. Colorectal cancer most commonly metastasizes to the liver via the portal circulation and subsequently to the lung [58,59]. Sarcoma preferentially metastasizes to the lung, reflecting its predominant hematogenous route of dissemination [60,61]. These nonrandom patterns of cancer metastasis are summarized in Table 1. Thus, successful metastasis depends on more than vascular delivery, but also on anatomical access and biological compatibility with the target organ microenvironment.

After arrest within the microcirculation, disseminated cancer cells must adhere to the endothelium, extravasate, survive in a distant tissue environment, evade immune surveillance, and adapt to organ-specific stromal, vascular, and metabolic conditions. Key mediators include chemokine pathways such as CXCR4–CXCL12 signaling in bone and bone marrow, CCR7–CCL21 signaling in lymphatic tissues [63], integrin-mediated interactions with extracellular matrix proteins, and metabolic programs that enable survival under hypoxia and nutrient limitation [5,50,56,64,65]. Tumor-derived factors and extracellular vesicles can also condition distant tissues before the arrival of cancer cells, establishing pre-metastatic niches that enhance subsequent colonization [21,23] as described above.

The present framework extends Paget’s Seed and Soil Hypothesis [1,2] by emphasizing that the arterial circulation and terminal microcirculation serve as the physiological delivery system connecting the seed to the soil. Thus, arterial dissemination is not a random scattering of cancer cells but a highly selective process shaped by vascular anatomy, microcirculatory dynamics, molecular recognition, and host tissue compatibility. Organ tropism emerges from the integration of these mechanical and biological factors and represents the final stage in the anatomical and physiological pathway linking the primary tumor to distant metastatic colonization.

The clinical patterns summarized here underscore a central principle of metastatic biology: dissemination follows anatomically defined routes, but successful colonization requires selective compatibility between disseminated cancer cells and permissive organ microenvironments, as mentioned above. These interactions are further shaped by clonal evolution, immune escape, cancer dormancy, spatial tissue organization, and therapeutic selection, which together determine whether disseminated cells remain dormant, are eliminated, or progress to clinically overt metastases.

## 7. Emerging Technologies for Monitoring and Modeling Cancer Dissemination

Liquid biopsy has emerged as a minimally invasive approach for detecting cancer-derived material in the circulation, including circulating tumor cells (CTCs), circulating tumor DNA (ctDNA), extracellular vesicles (including exosomes), and tumor-educated platelets. These biomarkers may originate from cancer cells entering the bloodstream either indirectly through lymphatic channels and the thoracic duct, or directly through post-capillary venules at the primary tumor site [8,11,65,66].

Because these biomarkers can be sampled serially, liquid biopsy provides a dynamic and quantitative assessment of tumor burden, clonal evolution, intratumoral heterogeneity, minimal residual disease, and impending recurrence, often preceding radiographic detection of metastatic disease [67,68,69]. Circulating tumor cell clusters exhibit substantially greater metastatic potential than single cells, underscoring the biological relevance of collective dissemination [70].

In parallel, artificial intelligence (AI), including machine learning and deep learning, enables the integration of histopathology, radiologic imaging, genomic sequencing, spatial transcriptomics, and liquid biopsy data to identify complex patterns associated with metastatic progression, therapeutic response, and recurrence [71,72]. AI-based image analysis can quantify tumor architecture, lymphatic vessel density, immune infiltration, and spatial relationships among cancer and stromal cells, while predictive models applied to ctDNA and CTC data may refine risk stratification and guide individualized treatment decisions [73,74,75]. These computational approaches are particularly relevant to studies of the SLN, where integration of spatial and molecular features may reveal early biomarkers of recurrence and response to immunotherapy.

While the present review focuses primarily on the anatomical and physiological routes of cancer dissemination, it does not fully address the extensive cellular and molecular heterogeneity of the tumor microenvironment that also influences metastatic progression. Recent advances in single-cell RNA sequencing, spatial transcriptomics, multiplex imaging, and spatial multiomic technologies have revealed remarkable diversity among immune, stromal, endothelial, and malignant cell populations within primary tumors, sentinel lymph nodes, and metastatic sites [76,77]. These approaches permit high-resolution mapping of cellular interactions and immune states that may influence whether disseminated cancer cells are eliminated, remain dormant, evade immune recognition and control, or successfully establish metastatic colonies. Emerging evidence further indicates that specific tumor microenvironment compositions may predict responsiveness to immunotherapy and contribute to differences in metastatic behavior among patients with otherwise similar clinicopathologic characteristics [76,77]. Future integration of single-cell and spatial multiomic analyses with anatomical models of dissemination may provide a more comprehensive understanding of how tumor microenvironment heterogeneity influences lymphatic and hematogenous metastatic routes while simultaneously identifying novel biomarkers and therapeutic vulnerabilities [76,77].

Together, liquid biopsy, artificial intelligence, single-cell technologies, and spatial multiomic approaches complement conventional anatomical staging by providing dynamic, quantitative, and increasingly predictive tools to detect minimal residual disease, characterize tumor microenvironment heterogeneity, monitor systemic dissemination, and model metastatic trajectories in individual patients. These emerging platforms offer the potential to integrate anatomical routes of spread with the biological ecosystems that regulate metastatic progression, immune interactions, and therapeutic response.

## 8. Clinical Implications and Integrative Conclusions

Cancer metastasis is the defining feature that distinguishes malignant from benign tumors and accounts for the majority of cancer-related deaths. This review presents a unified anatomical and physiological framework in which lymphatic and hematogenous dissemination are viewed as complementary and interconnected pathways within a continuous metastatic cascade [1,3].

In this model, the interstitial space serves as the central extracellular compartment linking the blood and lymphatic circulations. Cancer cells may enter highly permissive initial lymphatic capillaries and travel to the SLNs or undergo active intravasation through post-capillary venules. Tumor-derived cytokines, chemokines, and extracellular vesicles, together with cancer-associated lymphangiogenesis, condition the SLN and regional lymph nodes to form pre-metastatic niches that may eliminate disseminated cells, maintain dormancy, or promote metastatic expansion [21,23].

Cancer cells that survive these early stages of dissemination ultimately converge within the systemic venous circulation, undergo cardiopulmonary transit, and are redistributed through the arterial circulation to distant organs (Figure 1). At the microcirculatory site (Figure 2), the metarteriole, true capillary network, and post-capillary venule form a continuous microvascular unit that governs tumor cell arrest, extravasation, and entry into the interstitial space. Successful colonization depends on the compatibility of disseminated cancer cells with organ-specific vascular, stromal, immune, and metabolic microenvironments, thereby integrating the anatomical “delivery system” with Paget’s Seed and Soil Hypothesis [1,2,3,49].

Different patterns of cancer metastasis help explain why SLN biopsy is highly informative in melanoma [47] and breast cancer [48], why pulmonary surveillance is central in sarcoma, why the liver is the predominant first metastatic site in colorectal cancer, and why bone is a favored target in prostate cancer. Cancer metastasis patterns also identify therapeutic vulnerabilities involving lymphangiogenesis, endothelial adhesion, immune checkpoints, and organ-specific microenvironmental dependencies. Emerging technologies such as liquid biopsy and artificial intelligence further enable the dynamic detection of minimal residual disease and more accurate prediction of cancer recurrence.

This manuscript, presented as a review, offers a distinctive conceptual synthesis that integrates classical anatomy, microvascular physiology, lymphatic biology, tumor immunology, and clinical oncology into a dynamic model of cancer dissemination. This anatomical and physiological scaffold provides the foundation for subsequent manuscripts in this series addressing clonal evolution, spatial omics, cancer dormancy, artificial intelligence, and liquid biopsy, with the ultimate goal of improving early detection, risk stratification, and therapeutic intervention.

## Figures and Tables

**Table 1:** **Different Patterns of Metastasis in Selected Solid Cancers**. This table summarizes representative patterns of metastatic dissemination among selected solid cancers. Melanoma and breast cancer commonly involve sentinel or regional lymph nodes early, supporting the clinical importance of lymphatic mapping and nodal staging. Lung cancer frequently involves hilar or mediastinal lymph nodes and later systemic sites, including bone, brain, adrenal gland, and liver. Prostate cancer characteristically metastasizes to bone, especially the axial skeleton, whereas colorectal cancer commonly metastasizes to the liver through portal venous drainage and subsequently to the lung. In contrast, sarcoma most commonly disseminates hematogenously to the lung, while lymph-node metastasis is uncommon except in selected histologic subtypes.

Cancer Type	First Metastatic Niche	Later Metastatic Niches	Main Dissemination Route(s)	Predominant Diagnosis/Staging Emphasis
Melanoma	Sentinel/regional lymph nodes	Lung, liver, brain, bone, small bowel	Lymphatic spread with later systemic hematogenous dissemination	SLN status is a key staging and prognostic marker because of strong lymphatic tropism [53].
Breast cancer	Axillary sentinel/regional lymph nodes	Bone, lung, liver, brain	Lymphatic spread with later systemic hematogenous dissemination	SLN/axillary staging is important because of common lymphatic drainage; later metastasis follows organ-tropic dissemination [56].
Lung cancer	Hilar/mediastinal lymph nodes	Bone, brain, adrenal gland, liver	Lymphatic spread with systemic hematogenous dissemination	Staging includes mediastinal nodal evaluation and imaging for common distant sites, especially brain and adrenal involvement [62].
Prostate cancer	Pelvic lymph nodes	Bone, lymph nodes, lung, liver	Lymphatic spread with later systemic hematogenous dissemination	Bone imaging is important because prostate cancer characteristically metastasizes to the axial skeleton [35,57].
Colorectal cancer	Mesenteric lymph nodes	Liver, lung, peritoneum	Lymphatic spread and hematogenous spread through portal venous circulation	Liver surveillance is important because hepatic metastasis is common through portal venous drainage [58,59].
Sarcoma	Lung	Lung, bone, liver	Predominantly hematogenous; nodal spread is uncommon except in selected subtypes	Chest surveillance is central because lung metastasis predominates; nodal evaluation is selective and subtype-dependent [60,61].

## Data Availability

No new data were created or analyzed in this study. Data sharing is not applicable to this article.

## Abbreviations

The following abbreviations are used in this manuscript:

SLN	Sentinel Lymph Node
VEGF	Vascular Endothelial Growth Factor
VEGFR	Vascular Endothelial Growth Factor Receptor
CTCs	Circulating Tumor Cells
ctDNA	Circulating Tumor DNA
AI	Artificial Intelligence
TNF-α	Tumor Necrosis Factor-alpha
HEV	High Endothelial Venules
TME	Tumor Microenvironment
